# Functional neuroanatomy of reading in Czech: Evidence of a dual-route processing architecture in a shallow orthography

**DOI:** 10.3389/fpsyg.2022.1037365

**Published:** 2023-01-16

**Authors:** Marek Bartoň, Steven Z. Rapcsak, Vojtěch Zvončák, Radek Mareček, Václav Cvrček, Irena Rektorová

**Affiliations:** ^1^Applied Neuroscience Research Group, Central European Institute of Technology – CEITEC, Masaryk University, Brno, Czechia; ^2^Department of Neurology, University of Arizona, Tucson, AZ, United States; ^3^Department of Telecommunications, Faculty of Electrical Engineering and Communication, Brno University of Technology, Brno, Czechia; ^4^Institute of the Czech National Corpus, Charles University, Prague, Czechia; ^5^International Clinical Research Center, ICRC, St. Anne’s University Hospital and Faculty of Medicine, Masaryk University, Brno, Czechia

**Keywords:** fMRI, lexical-semantic, phonology, reading, shallow orthography, visual word form area, VWFA

## Abstract

**Introduction:**

According to the strong version of the orthographic depth hypothesis, in languages with transparent letter-sound mappings (shallow orthographies) the reading of both familiar words and unfamiliar nonwords may be accomplished by a sublexical pathway that relies on serial grapheme-to-phoneme conversion. However, in languages such as English characterized by inconsistent letter-sound relationships (deep orthographies), word reading is mediated by a lexical-semantic pathway that relies on mappings between word-specific orthographic, semantic, and phonological representations, whereas the sublexical pathway is used primarily to read nonwords.

**Methods:**

In this study, we used functional magnetic resonance imaging to elucidate neural substrates of reading in Czech, a language characterized by a shallo worthography. Specifically, we contrasted patterns of brain activation and connectivity during word and nonword reading to determine whether similar or different neural mechanisms are involved. Neural correlates were measured as differences in simple whole-brain voxel-wise activation, and differences in visual word form area (VWFA) task-related connectivity were computed on the group level from data of 24 young subject. Trial-to-trial reading reaction times were used as a measure of task difficulty, and these effects were subtracted from the activation and connectivity effects in order to eliminate difference in cognitive effort which is naturally higher for nonwords and may mask the true lexicality effects.

**Results:**

We observed pattern of activity well described in the literature mostly derived from data of English speakers – nonword reading (as compared to word reading) activated the sublexical pathway to a greater extent whereas word reading was associated with greater activation of semantic networks. VWFA connectivity analysis also revealed stronger connectivity to a component of the sublexical pathway - left inferior frontal gyrus (IFG), for nonword compared to word reading.

**Discussion:**

These converging results suggest that the brain mechanism of skilled reading in shallow orthography languages are similar to those engaged when reading in languages with a deep orthography and are supported by a universal dual-pathway neural architecture.

## 1. Introduction

Overt reading is a process related to coordinated left-lateralized neural network activity comprising sensory, language, attentional, and articulatory motor components. The sensory component consists of basic visual perception followed by higher visual processing when particular elements of the text are discriminated. A critical region involved in this function is the visual word form area (VWFA) located in the ventral occipitotemporal cortex (Cohen et al., 2002). It has been postulated that this region is involved in discrimination of letters, and letter combinations, and in processing strings of letters with and without lexical value (Dehaene et al., 2005). It contains neural representations of whole words (Glezer et al., 2009; Schuster et al., 2016), hence playing a crucial role in general orthographic processing (Glezer et al., 2016; Fischer-Baum et al., 2017) and in modality-independent lexical access (Sebastian et al., 2014). Anatomical (Botthali et al., 2014; Lerma-Usabiaga et al., 2018) and resting-state functional (Stevens et al., 2017) connections of the VWFA to language-specific regions predetermine its important role in processing and transmitting information from lower visual regions to language areas. It has been suggested that the VWFA cortex was evolutionarily tuned to play a role in language functions such as object naming and lip reading (Ghuman and Fiez, 2018), rendering it a suitable neural substrate for specialization during reading acquisition (Saygin et al., 2016).

The orthographic information identified in the VWFA is then processed in language regions (Woollams et al., 2018) involved in orthography-to-phonology (print-to-sound) conversion. Based on observations predominantly of English-speaking subjects, this conversion of written to spoken forms is computed *via* the interplay of two neural circuits, the ventral lexical-semantic and dorsal sublexical pathways, according to the dual-route hypothesis (Coltheart et al., 2001; Richlan et al., 2009; Grainger and Ziegler, 2011). The lexical-semantic pathway comprises extrasylvian regions – the triangular and orbital parts of the inferior frontal gyrus, temporal pole, middle/inferior temporal gyri, anterior fusiform gyrus and angular gyrus (Price, 2012; Rapcsak and Beeson, 2015) – and it has been implicated in quickly addressing the phonology of whole word forms in relation to word meaning (Taylor et al., 2015). The phonological/sublexical pathway encompasses perisylvian regions – the opercular part of the inferior frontal gyrus (IFGop), superior temporal gyrus/sulcus (STG/STS), precentral gyrus, insula and inferior parietal regions (Price, 2012, Rapcsak and Beeson, 2015) playing a crucial role in the serial phoneme to grapheme conversion that is necessary for processing novel nonwords. Neuroimaging studies focusing on the word and pseudoword reading (almost exclusively examining the deep orthography languages) clearly showed the distinction between involvement of the lexical-semantic vs. sublexical pathways (Binder et al., 2005; Taylor et al., 2013); essentially the former relies on interactions between the VWFA and ventral language pathway regions/semantic network for word reading and latter on interactions between the VWFA and dorsal language pathway/phonological network for nonword reading.

According to the orthographic depth hypothesis (ODH) (Katz and Frost, 1992; Frost, 2005), reliance on the lexical-semantic vs. sublexcial pathways in reading is influenced by the predictability and complexity of print-to-sound correspondences (Schmalz et al., 2015) of a given writing system. In deep/opaque orthographies, the lexical-semantic pathway is the dominant procedure used for reading familiar words and is essential to correctly process irregular words that have atypical or exceptional letter-sound mappings. By contrast, the sublexical route is used primarily to read unfamiliar nonwords. According to the “strong version” of ODH, in shallow/transparent orthographies with predictable letter-sound correspondences correct pronunciations for both words and nonwords could be computed *via* the sublexical pathway. In contrast to this theoretical assumptions, there is behavioral evidence (Raman et al., 1996; Pagliuca et al., 2008; Marcolini et al., 2009; Difalcis et al., 2018; Ripamonti et al., 2018), neuropsychological evidence (Ardila and Cuetos, 2016), modelling (Seidenberg, 2011; Kapnoula et al., 2017), and neuroimaging (Ischebeck et al., 2004; Danelli et al., 2015; Rueckl et al., 2015; Marinelli et al., 2016; Protopapas et al., 2016) suggesting that even in transparent orthographies the lexical-semantic processes are used for skilled reading of words. This evidence contradicts the “strong version” of ODH suggesting that there are distinct lexical-semantic and sublexical pathways in both shallow and deep orthographies which suggest that reading in all languages is supported by a neural network characterized by a universal dual-pathway architecture, and orthographic depth may influence the division of labor between the lexical-semantic and sublexical pathways (Paulesu et al., 2000; Das et al., 2011; Cherodath and Singh, 2015; Mei et al., 2015; Oliver et al., 2017).

Language neural processes are accompanied by bilateral fronto-insular-parietal multiple-demand system (MDS) activity which plays a role in domain-general processes (Duncan, 2010; Fedorenko et al., 2013). These consist of cognitive/attentional control supported by the interplay of the frontoparietal control network [FPN (Paulesu et al., 2000; Gao and Lin, 2012; Dixon et al., 2018)] with the cingulo-opercular network [(Dosenbach et al., 2007; Ihnen et al., 2015)], and the attention and working memory functions of the dorsal attention network [DAN (Ihnen et al., 2015; Majerus et al., 2018)]. The MDS activity is not directly involved in language processing but rather reflects general cognitive effort (Blank and Fedorenko, 2017; Khachouf et al., 2017) proportionate to task difficulty. In reading studies, this difficulty corresponds (in addition to other possible factors) to stimulus novelty which is higher for nonwords than for known words (Taylor et al., 2014). The MDS activity is linked to the following bilateral areas: middle and inferior frontal gyri, anterior insula, medial superior frontal cortices (i.e., supplementary and presupplementary motor areas), dorsal anterior cingulate, and superior and inferior parietal lobules. Activation of language-related and MDS networks is followed by the articulatory activity which is executed *via* speech motor network [orofacial motor cortex in precentral gyrus, supplementary motor area in medial frontal gyrus, putamen, and cerebellum (Price, 2012)].

During reading (as well as during other tasks requiring increased externally oriented attention as compared to rest), the default mode network (DMN) (Raichle and Snyder, 2007) activity decreases (Mineroff et al. 2018; Branco et al., 2020). The DMN encompasses the (bilateral) precuneus/posterior cingulate cortex, angular gyri, ventromedial prefrontal/anterior cingulate cortex, dorsal frontal cortices, lateral temporal cortices, hippocampus, and perihippocampal structures. The DMN as a unit has traditionally been implicated in semantic processing (Binder et al., 2009), and its activation level is higher when reading words compared to reading nonwords. It has been is hypothesized that the DMN activity level is determined by a combination of deactivation that is proportional to task difficulty and semantic-related activation that reduces the deactivated state to some degree (Zhang et al., 2016) – the left angular gyrus, a part of the lexical-semantic language pathway also belongs to the DMN.

Our study focuses on the functional neuroimaging of neural substrates involved in reading aloud in Czech – an alphabetical language with a shallow orthography. More specifically, we assess the involvement of the dorsal and ventral language pathways linked to lexicality manipulation – differences in word and nonword reading. Such experiments are rather marginally represented in literature the (Taylor et al., 2015), as most research focused on the neural substrates of reading has been done with English (Share, 2008). In our blood-oxygen-level-dependent functional magnetic resonance imaging (BOLD fMRI) experiment, we employ a classical analysis of activations and task-related VWFA connectivity: the generalized psychophysiology interaction method [gPPI (McLaren et al., 2012; O’Reilly et al., 2012)]. To our knowledge, it is the first study of neural correlates of lexicality effects in Czech (as a shallow orthography). We additionaly aim at controlling for the difficulty effects approximated with response times (RTs) to separate only the differences in language-related processing beyond the increased task demands (i.e., trial-to-trial cognitive effort) that are naturally greater for nonword reading than to word reading. Based on the literature in English speakers, if reading in Czech is also supported by a dual-pathway, we hypothesize pattern stronger activation for words than nonwords in ventral language pathway and DMN, and for nonwords than for words in the dorsal language pathway. For the VWFA connectivity, we hypothesize increased functional interaction with components of the dorsal pathway during nonword compared to word reading and with components of the ventral pathway and DMN during word compared to nonword reading.

## 2. Materials and methods

### 2.1. Block-design reading task

#### 2.1.1. Subjects

Thirty subjects were enrolled in this study. None of them reported any previous neurologic or psychiatric disorders. All subjects gave their written informed consent, and the study was approved by the local ethics board. Six subjects were excluded because of the insufficient quality of their physiological recording, poor fMRI data coverage or excessive dropouts, or low task performance. The mean ± SD age of the final cohort of 24 subjects (12 females) was 24.3 ± 3.1 years (max. 30 years). All subjects had successfully completed at least a high school level of education.

#### 2.1.2. Tasks and experiment stimuli

The fMRI task consisted of the overt reading of Czech words and nonwords. A total of 40 words, 40 nonwords, and 40 control visual stimuli (nonflickering checkerboards) were presented in the experiment part. All words were high-frequency two-syllable nouns. The frequency range of words was 136.29 ppm to 801.01 ppm (mean 259.86, std. 141.96). The length range of words was 4 to 7 letters (mean 5.1, std. 0.81) All nonwords were pronounceable, created by shifting syllables or letter groups in the real word stimuli used in the experiment and having the same lengths. Prior to the main experiment part, a short training session was administered with 20 words, 20 nonwords, and 20 checkerboards (words and nonwords were different from those in the main experiment part), allowing the subjects to become accustomed to the MRI environment and to practice overt reading with minimal head motion. All stimuli were presented visually *via* a mirror mounted to the head coil. Subject responses were collected using an optoacoustic microphone during the reading task in order to control the task performance and to estimate reading response times. All words were two-syllable, very frequent nouns with pronunciation allowing the reliable detection of overt reading onsets. All nonwords were pronounceable, created by shifting syllables or letter groups.

The particular trials were organized into blocks containing five similar trials with instruction to “read these words,” “read these nonwords,” and “view the checkerboards” at the beginning of particular blocks. The order of blocks was pseudorandomized (with this repeating pattern: 1. words/nonwords, 2. nonwords/words, 3. baseline). Along with the reading, a spelling task was administered with the same sets of words and nonwords, but this text is only focused only on reading task. The order of reading and spelling tasks was balanced across subjects.

### 2.2. Data acquisition

Subjects were scanned with the 3 T Siemens Prisma MR scanner. The measurement consisted of four functional runs (two training and two experimental task runs). Scanning fMRI parameters were set: TR = 704 ms, TE = 35 ms, flip angle = 46°, voxel size = 3 × 3 × 3 mm^3^, no gap between slices, 44 transversal slices with in-plane matrix size 64 × 64 voxels. A multiband factor of 4 and a PAT factor of 2 were used. Plane orientation was approximately according to the AC-PC direction; acquisition volume covered almost the whole brain excluding the inferior part of the cerebellum. In total, 530 and 1,040 volumes per training and experimental runs were collected, respectively. The high-resolution anatomical T1-weighted images were acquired before functional scanning using the MP-RAGE sequence with 224 sagittal slices, matrix size 240 × 224, voxel size 1 × 1 × 1 mm^3^, TR = 2,300 ms, TE = 2.33 ms, flip angle = 8°. The ECG and breathing signal data were recorded simultaneously during functional measurement using the MR compatible EEG/ExG system (Brain Products, Germany). Two ECG curves were measured with electrodes placed in pairs under the left clavicle and on the lateral side of the thorax. A pneumatic belt was placed on the upper part of the abdomen to capture breathing-linked motion. The overt speech was recorded using optoacoustic microphone and Audacity software for the reaction time estimate.

### 2.3. Data pre-processing

The data was preprocessed in a routine way in SPM12 toolbox. The preprocessing involved the realign&unwarp function and correction for physiological pulse and breathing artifacts with RETROICOR (Glover et al., 2000), normalization to MNI space using a coregistered anatomical image, and spatial smoothing with a 5 mm isotropic Gaussian kernel. Data preprocessed in this way were used for activation analysis.

The additional preprocessing for connectivity analysis (Barton et al., 2015, 2019) consisting of ICA-based denoising was used. The ICA decomposition was done for both task data concatenated in the GIFT toolbox with minimum descriptive length criterion (Li et al., 2007) ensuring the good stability of the estimated components. Artificial components were selected based on their spatial patterns (location in liquor and/or in large vessels) and their time-courses were regressed out of the data.

### 2.4. Speech recordings processing and evaluation of RTs and behavioral performance

All speech recordings were processed in WaveSurfer. After an automated denoising procedure to suppress MRI scanner noise, speech onsets related to reading both words and nonwords were manually detected. The mean RTs of words and nonwords per subject were compared with a paired t-test, and the significance was assessed at a threshold of *p* = 0.05.

After the scanning session, the subjects performed the same reading task (with the same word and nonword stimuli) outside the scanner in order to assess individual task accuracy since the recording quality was sufficient to detect reading onsets but not suitable to assess accuracy of all items in all subjects. For reading accuracy comparison between words and nonwords, paired t-test was used and the significance was assessed at threshold p = 0.05.

### 2.5. Activation analysis

A general linear model was used with all three conditions modeled as boxcar functions (one for each block type) convolved with the standard canonical hemodynamic response function. An additional parametric modulation regressor representing all RTs for word and nonword trials was implemented as in the study by Taylor et al. (2014) in order to capture difficulty effects making it possible to assess the between-condition activation differences over and above RT.

Group statistics were computed with regressors representing sex and task order (representing the current reading task and the writing task that is not included in this study). All resulting statistical maps were thresholded with an initial voxel-wise threshold of *p* = 0.005 uncorrected for multiple comparisons and subsequently with p = 0.05 FWE corrected on the cluster level. The description of the anatomical localization of significant activation effects was carried out by an in-house Matlab script using the automated anatomical labeling (AAL) atlas of gray matter (Tzourio-Mazoyer et al., 2002).

### 2.6. Psychophysiological interactions analysis

The generalized form of psychophysiological interaction analysis (McLaren et al., 2012) (gPPI) was employed to assess the between-condition differences of VWFA connectivity. A seed signal was extracted from the ROI including voxels with positive reading words>baseline effect in a 6 mm sphere centered at the individual (subject-specific) coordinates of the VWFA in order to correctly capture the subtle individual differences in VWFA location (Glezer and Riesenhuber, 2013) that are crucial for correct VWFA connectivity analysis (Caffarra et al., 2021). These coordinates were estimated as in the study by Purcell et al. (Purcell et al., 2017) from the nearest local activation maxima to [−43, −55, −17] (*x*, *y*, *z* in mm in MNI space) for words>baseline comparison. This anterior part of the VWFA is involved in lexical processing (Lerma-Usabiaga et al., 2018; Caffarra et al., 2021).

The gPPI model included three condition-related interaction gPPI terms (for words and nonwords, these gPPI terms were orthogonalized to the RT gPPI regressor), together with block-related modeled activations as regressors of no interest. Additionally, the RT gPPI interaction term for word and nonword trials to regress out the RT-related connectivity modulation and RT-modulated word and nonword activation time course were added as regressors of no interest to capture difficulty effects.

Group statistics were computed with regressors representing sex, task order, and individual ROI size. All resulting statistical maps were thresholded with an initial voxel-wise threshold of *p* = 0.005 uncorrected for multiple comparisons and subsequently with *p* = 0.05 FWE corrected on the cluster level. The description of the anatomical localization of significant connectivity effects was carried out by the in-house Matlab script using AAL atlas of gray matter (Tzourio-Mazoyer et al., 2002).

## 3. Results

### 3.1. Behavioral performance – RTs

The average RT for word stimuli was 806.52 ± 14.09 ms (mean ± standard deviation), and the average RT for nonword stimuli was 956.81 ± 150.85 ms. The difference between word and nonword RTs was statistically significant, *p* < 0.05 (*p* = 2.23*10^8^).

### 3.2. Behavioral performance after the scanning session

All words were read correctly in all subjects, i.e., reaching 100% accuracy; nonwords were read with 99.98% ± 0.03% (mean ± standard deviation) accuracy. The difference in reading accuracy between the word and nonword stimuli was statistically significant at p < 0.05 (*p* = 1.56*10^4^).

### 3.3. Activation analysis

**Figure 1:**
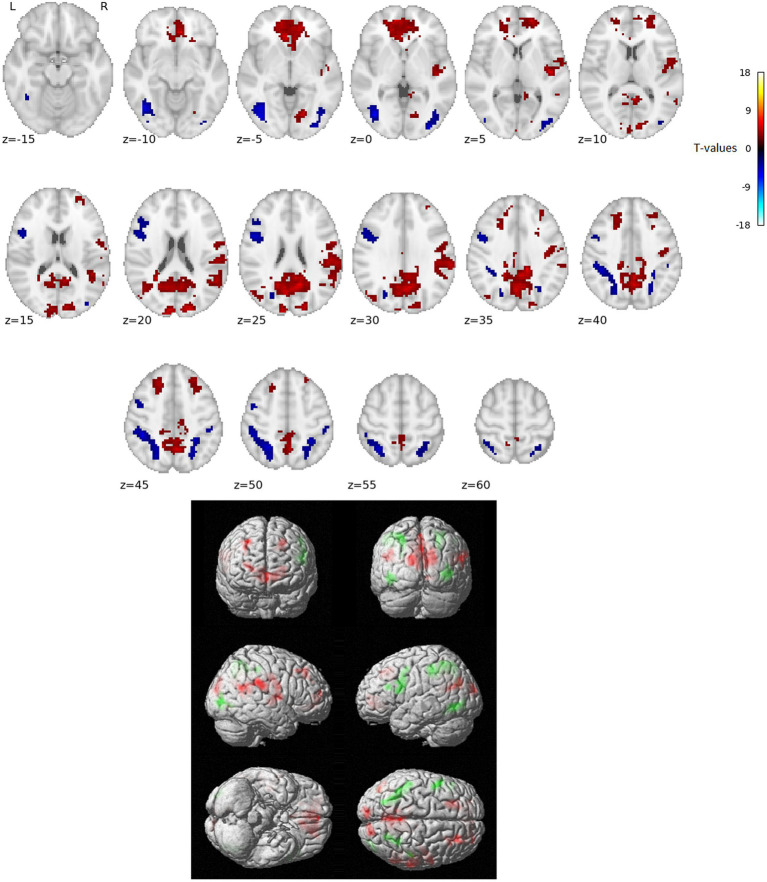
Statistically significant group effect of reading words vs. reading nonwords after RT effects were regressed out (*T*-statistics). Red indicates neural activity stronger for reading words over and above RT effects, blue/green indicates neural activity stronger for reading nonwords over and above RT effects. Thresholded with *p* = 0.005 uncorrected for multiple comparisons at the voxel level and subsequently with *p* = 0.05 FWE corrected at the cluster level. Positions of slices in the *z*-axis in MNI space are indicated below the slices; L and R mark the left and right side, respectively.

The activation analysis results comparing word reading vs. nonword reading after regressing out the RT effects (i.e., over and above RTs) are in Figure 1 and Table 1.

**Table 1:** Listing of anatomical structures with statistically significant group effect of reading words vs. reading nonwords after RT effects were regressed out –(a) words>nonwords over and above RTs; (b) words<nonwords over and above RTs.

(a) Activation over and above RT: Words > Nonwords					
*k*	max *T*	*X*	*y*	*z*	Anatomical label
29	4.04	57	−1	26	R Precental gyrus
82	4.37	−24	50	−1	L Superior frontal gyrus
66	5.51	18	53	5	R Superior frontal gyrus
75	4.28	−21	32	44	L Middle frontal gyrus
81	4.34	27	17	38	R Middle frontal gyrus
95	4.81	54	−25	23	R Rolandic operculum
12	4.08	6	23	−7	R Olfactory cortex
20	5.89	−12	53	−1	L Superior frontal gyrus, medial
63	4.16	15	53	5	R Superior frontal gyrus, medial
42	4.72	−12	50	−4	L Superior frontal gyrus, medial - orbital
65	5.79	6	38	−10	R Superior frontal gyrus, medial - orbital
42	4.83	42	−13	5	R Insula
94	5.15	−6	29	−7	L Anterior cingulate and paracingulate gyri
38	5.78	6	38	−7	R Anterior cingulate and paracingulate gyri
82	4.06	0	−46	35	L Median cingulate and paracingulate gyri
112	6.09	12	−37	35	R Median cingulate and paracingulate gyri
53	4.11	0	−46	32	L Posterior cingulate gyrus
27	4.85	6	−49	26	R Posterior cingulate gyrus
35	4.20	−6	−64	20	L Calcarine fissure and surrounding cortex
41	4.54	9	−88	11	R Calcarine fissure and surrounding cortex
137	5.39	−9	−91	23	L Cuneus
94	5.22	15	−91	20	R Cuneus
53	5.10	18	−70	−4	R Lingual gyrus
55	5.41	−9	−91	20	L Superior occipital gyrus
51	5.44	18	−91	20	R Superior occipital gyrus
30	3.90	−42	−76	29	L Middle occipital gyrus
66	4.26	60	−1	23	R Postcentral gyrus
146	4.85	63	−31	29	R Supramarginal gyrus
19	3.92	−39	−61	23	L Angular gyrus
12	3.54	57	−61	26	R Angular gyrus
328	5.58	−3	−64	32	L Precuneus
325	5.88	12	−58	26	R Precuneus
21	5.41	45	−16	5	R Heschl gyrus
86	4.96	45	−19	2	R Superior temporal gyrus
34	3.94	−42	−61	20	L Middle temporal gyrus
49	4.60	54	−52	20	R Middle temporal gyrus
(b) Activation over and above RT: Words < Nonwords					
*k*	max *T*	*X*	*y*	*z*	Anatomical Label
108	4.64	−51	5	26	L Precental gyrus
86	5.21	−51	11	17	L Inferior frontal gyrus, opercular part
55	3.92	−45	23	20	L Inferior frontal gyrus, triangular part
14	3.70	−21	−70	38	L Superior occipital gyrus
25	3.96	24	−64	44	R Superior occipital gyrus
57	6.52	−39	−73	−1	L Middle occipital gyrus
76	5.66	33	−91	−1	R Middle occipital gyrus
56	6.29	−39	−67	−7	L Inferior occipital gyrus
22	5.00	36	−85	−7	R Inferior occipital gyrus
16	4.61	−42	−52	−13	L Fusiform gyrus
23	5.40	−42	−37	47	L Postcentral gyrus
102	5.44	−30	−58	56	L Superior parietal lobule
56	3.85	24	−58	50	R Superior parietal lobule
210	5.65	−42	−37	44	L Inferior parietal lobule
44	4.63	30	−49	50	R Inferior parietal lobule
23	5.65	−42	−61	−10	L Inferior temporal gyrus

For every structure, number of voxels with significant effect (*k*) and peak *T*-statistics (max *T*) with corresponding *x*, *y*, *z* MNI coordinates are reported. Thresholded with *p* = 0.005 uncorrected for multiple comparisons at the voxel level and subsequently with *p* = 0.05 FWE corrected at the cluster level.

### 3.4. Psychophysiological interaction analysis

**Figure 2:**
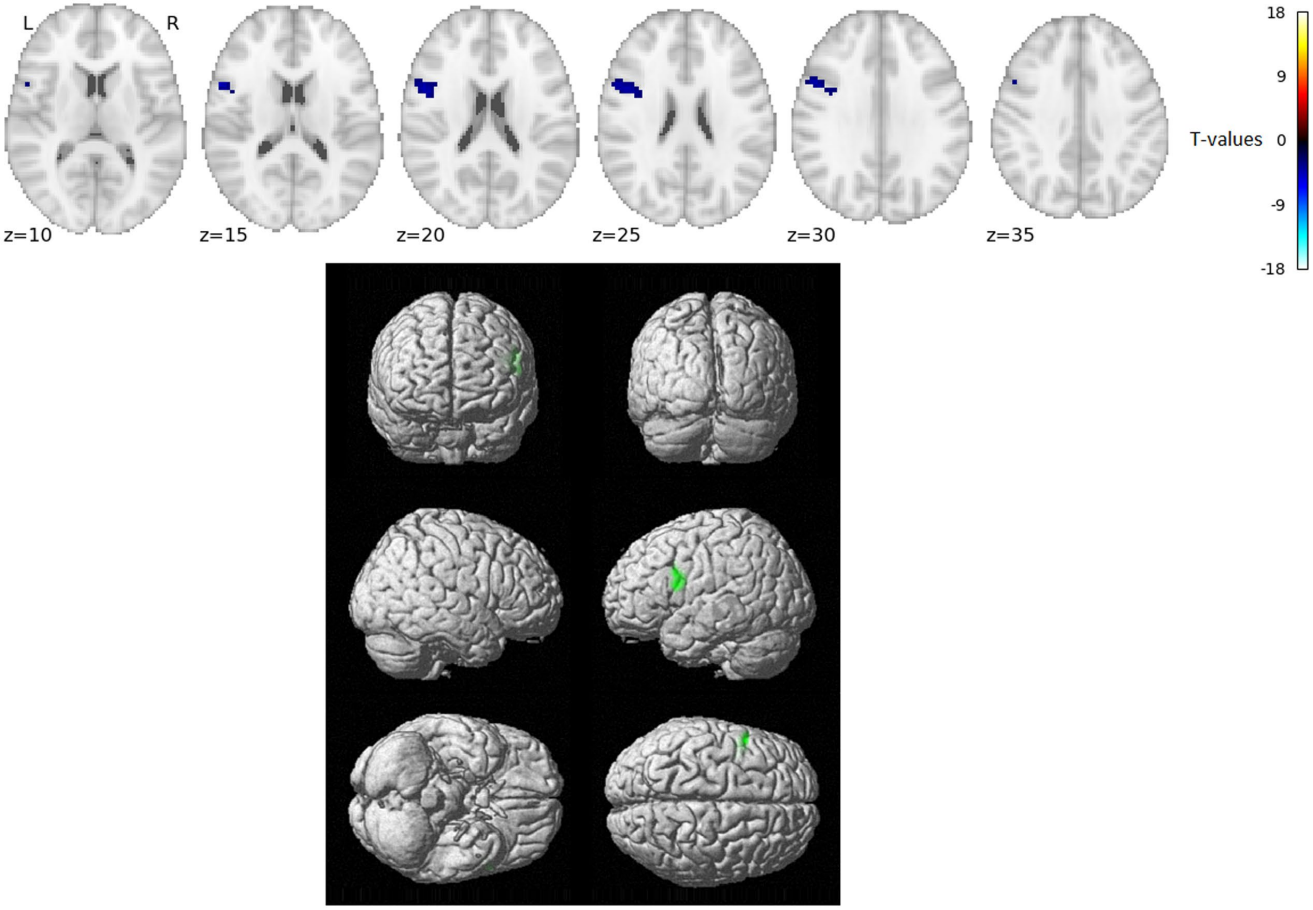
Statistically significant group effect of VWFA gPPI connectivity – reading words vs. reading nonwords after RT-related connectivity modulation effects were regressed out (*T*-statistics). No VWFA connectivity stronger for reading words over and above RT effects was observed, blue/green indicates VWFA connectivity stronger for reading nonwords over and above RT effects. Thresholded with *p* = 0.005 uncorrected for multiple comparisons at the voxel level and subsequently with *p* = 0.05 FWE corrected at the cluster level. Positions of slices in the z-axis in MNI space are indicated below the slices; L and R mark the left and right side, respectively.

The VWFA gPPI connectivity analysis results for comparing word reading and nonword reading after regressing out the RT effects (i.e., over and above RTs) are shown in Figure 2 and Table 2.

**Table 2:** Listing of anatomical structures with statistically significant group effect of VWFA gPPI connectivity – reading words vs. reading nonwords after RT-related connectivity modulation effects were regressed out (words<nowords above and over RTs only, no effects of words>nonwords above and over RTs were observed).

VWFA gPPI connectivity over and above RT: Words < Nonwords					
*k*	max *T*	*x*	*y*	*z*	Anatomical Label
76	4.98	−57	11	17	L Inferior frontal gyrus, opercular part

For a given structure, number of voxels with significant effect (*k*) and peak *T*-statistics (max *T*) with corresponding *x*, *y*, *z* MNI coordinates are reported. Thresholded with *p* = 0.005 uncorrected for multiple comparisons at the voxel level and subsequently with *p* = 0.05 FWE corrected at the cluster level.

## 4. Discussion

Recent functional imaging research on neural substrates of language processing has shown a basic language neural network architecture that is similar across wide range of languages (Malik-Moraleda et al., 2022). However, many linguistic variables such as orthographic depth may play roles in variations of particular language-related processes. This topic is underrepresented in literature, since the majority of this research is conducted with English speakers, reflecting a deep orthography language.

In the present study, we used fMRI in order to identify neural correlates of reading in Czech, i.e., a language with shallow orthography. Lexical (words) and nonlexical (nonwords) stimuli were administered to uncover possible lexicality effects linked to different engagements of the sublexical and lexical-semantic language pathways. Comparison of activation between words and nonwords after regressing out the effects of RTs revealed higher activity for words, especially in regions of the DMN: posterior cingulate cortex/precuneus, anterior cingulate cortex/ventromedial prefrontal cortex, dorsal prefrontal cortex, bilateral angular gyrus, and bilateral middle temporal gyrus (with more right-lateralized temporo-parietal activation). The DMN word>nonword activation over and above RT effects may suggest automatic (Vatansever et al., 2017) associative internally-oriented processing linked to word meaning (Binder et al., 2009), thus supporting lexical/semantic processing, together with lexical pathway regions (angular and middle temporal gyri) engagement. The right temporoparietal cortex has been implicated in reflexive attentional orienting processes linked to lexical reading (Ekstrand et al., 2019). The nonword>word activation over and above RT effects was located in the VWFA, bilateral parietal DAN regions, triangular and opercular parts of the left inferior frontal gyrus, and left precentral gyrus. This different nonword processing compared to word processing reflects the decoding of unfamiliar combinations of letters followed by sublexical pathway engagement. As the triangular part of the IFG is part of lexico-semantic pathway, its activity may reflect the automatic retrieval of stored phonological word forms with similar combination of letters and the inhibition of such incorrect responses. The observation of parietal regions activity surviving correction for difficulty effects is consistent with results from study by Taylor et al. (2014) and may relate to efficient discrimination of pseudowords with no semantic impact. The results of VWFA connectivity for nonwords>words contrast after controlling for RT effects revealed only one cluster in the IFGop while no results were detected for word>nonwords contrast. The IFGop plays an important role in phonological processing (as a part of the sublexical pathway), and its damage results in broad phonological deficits including impaired nonword reading (Fiez et al., 2006).

We did not directly compare the neural substrates of language processing between groups of subjects with native languages differing in orthographic depth. This has been done in previous research inspecting a hallmark of reading proficiency – speech-print convergence of neural activity – in adults (Rueckl et al., 2015) and seven-year-old children (Chyl et al., 2021). Although these two fMRI studies did not study lexicality effects, both of them concluded that regardless of the orthographic depth, skilled reading is supported by a neural network with universal topology that varies only to a minor extent in the activation of language pathways (i.e., a slight shift to dorsal/phonological activations in shallow orthography languages, and more pronounced ventral/lexical-semantic activations in deep orthography languages). Paulesu et al. (2000) studied neural substrates of word and nonword reading in English (deep orthography) and Italian (shallow orthography) readers. The conclusion of the study also supported the universality of the reading network across both languages when comparing nonword and word reading. Again, there was a slight shift towards dorsal/phonological activations in Italian (shallow orthography) for word and nonword reading manifesting as stronger activation in left superior temporal cortex associated with phoneme processing, and ventral/lexical-semantic activations (in left posterior inferior temporal cortex and anterior IFG, i.e., areas implicated in retrieval of whole words) in English for nonword reading. Unfortunately, direct comparisons of lexicality effects between the two languages were not reported in this study (and measures of task difficulty/cognitive effort effects were not controlled for). Nevertheless, at least for English alone (i.e., without a direct comparison to other languages), lexicality effects with task difficulty effect subtraction were reported in the literature (Taylor et al., 2014) with analogical results to the current study – words compared to nonwords over and above RT effects activated more left angular and middle temporal gyri (as a parts of the ventral language pathway), whereas nonwords compared to words over and above RT effects activated more left-lateralized IFGop, precentral gyrus, insula and supramarginal gyrus (as a parts of dorsal language pathway).

Concerning the limitations of our study, it is important to note that when using fMRI, there is decreased sensitivity (and possibly a complete drop-out of the signal) to properly scan ventral part of the lexical pathway in particular (i.e., inferior temporal cortex) (Ojemann et al., 1997; Visser et al., 2010), so the sensitivity to capture lexicality effects in the lexical pathway is rather low. This may be the reason for the fact that there was no evidence of increased VWFA connectivity with ventral pathway for words, so this connectivity analysis provided rather partial support – i.e., only for nonwords>words – for the hypothesis of differential VWFA connectivity with dorsal vs. ventral pathway regions during words vs. nonword reading.

Taken together, our results show that there are differences in patterns of activation and connectivity for words vs. nowords in Czech which is consistent with the findings in English and other languages (Taylor et al., 2013), arguing against the strong version of the ODH.

## 5. Conclusion

This study provides insights into the functional neuroanatomy of reading in a shallow orthography. The results present lexicality effects as shown by a comparison between word and noword reading, i.e., the different engagement of the lexical/semantic and sublexical phonological pathways involved in orthography to phonology conversion. The lexicality effects – after controlling for task difficulty effects – were observed as distinct patterns of activation and VWFA connectivity. These converging results suggest that the neural pathways of skilled reading in a shallow orthography exploit similar mechanisms as reading in deep orthography in terms of print-to-speech conversion which is consistent with universal dual-pathway architecture. The result is clinically relevant and may be exploited in rehabilitation of speech deficits in languages with both shallow and deep orthographies.

## Data availability statement

The raw data supporting the conclusions of this article will be made available by the authors, without undue reservation.

## Ethics statement

The studies involving human participants were reviewed and approved by the Research Ethics Committee of Masaryk University. The patients/participants provided their written informed consent to participate in this study.

## Author contributions

MB and SR designed the experiment, performed the data analysis, interpreted the data, and wrote the manuscript. VZ performed the analysis of audio recordings and the response time detection. RM contributed to the data analysis. VC contributed to the experimental stimuli selection. IR contributed to the manuscript writing and supervised the whole study. All authors contributed to the article and approved the submitted version.

## Funding

This work was supported by the European Union’s Horizon 2020 research and innovation program under the Marie Skłodowska-Curie grant agreement no. 734718 (CoBeN), by the project LX22NPO5107 (MEYS): Funded by European Union – Next Generation EU, and by the Czech Agency for Health Research No. NV18-04-00346.

## Conflict of interest

The authors declare that the research was conducted in the absence of any commercial or financial relationships that could be construed as a potential conflict of interest.

## Publisher’s note

All claims expressed in this article are solely those of the authors and do not necessarily represent those of their affiliated organizations, or those of the publisher, the editors and the reviewers. Any product that may be evaluated in this article, or claim that may be made by its manufacturer, is not guaranteed or endorsed by the publisher.
